# Supplementary material for: Canagliflozin Modulates Hypoxia-Induced Metastasis, Angiogenesis and Glycolysis by Decreasing HIF-1α Protein Synthesis via AKT/mTOR Pathway
Source: Int J Mol Sci. 2021 Dec 11;22(24):13336. doi: 10.3390/ijms222413336 (PMC8704642; doi:10.3390/ijms222413336)
Supplement: Supplementary file 1 [file ijms-22-13336-s001.zip › supplementary materials-Fig. S1-S6.pptx]

## Slide 1
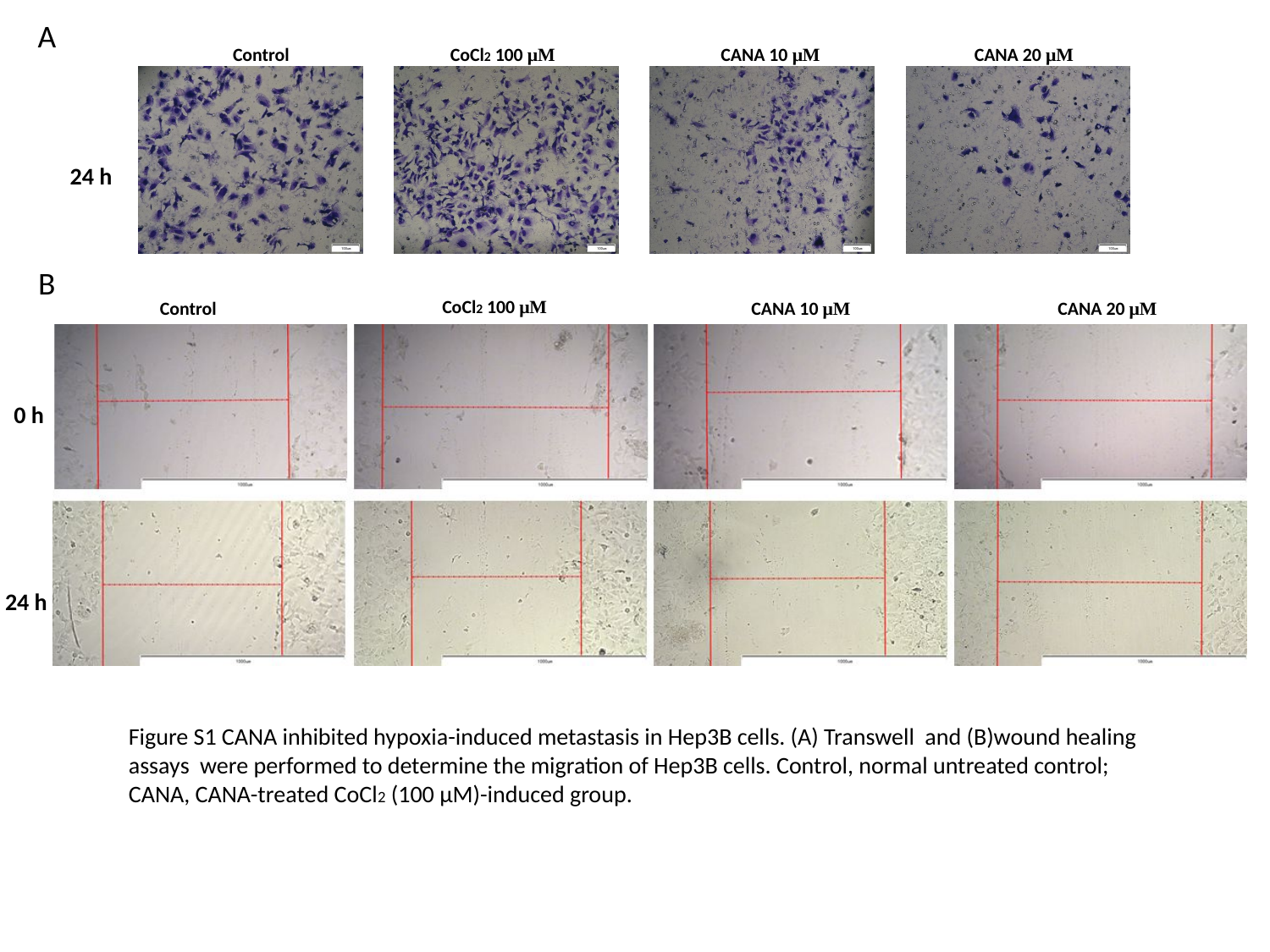

A
CANA 20 μM
CoCl2 100 μM
CANA 10 μM
Control
24 h
B
CoCl2 100 μM
Control
CANA 20 μM
CANA 10 μM
0 h
24 h
Figure S1 CANA inhibited hypoxia-induced metastasis in Hep3B cells. (A) Transwell and (B)wound healing assays were performed to determine the migration of Hep3B cells. Control, normal untreated control; CANA, CANA-treated CoCl2 (100 μM)-induced group.

## Slide 2
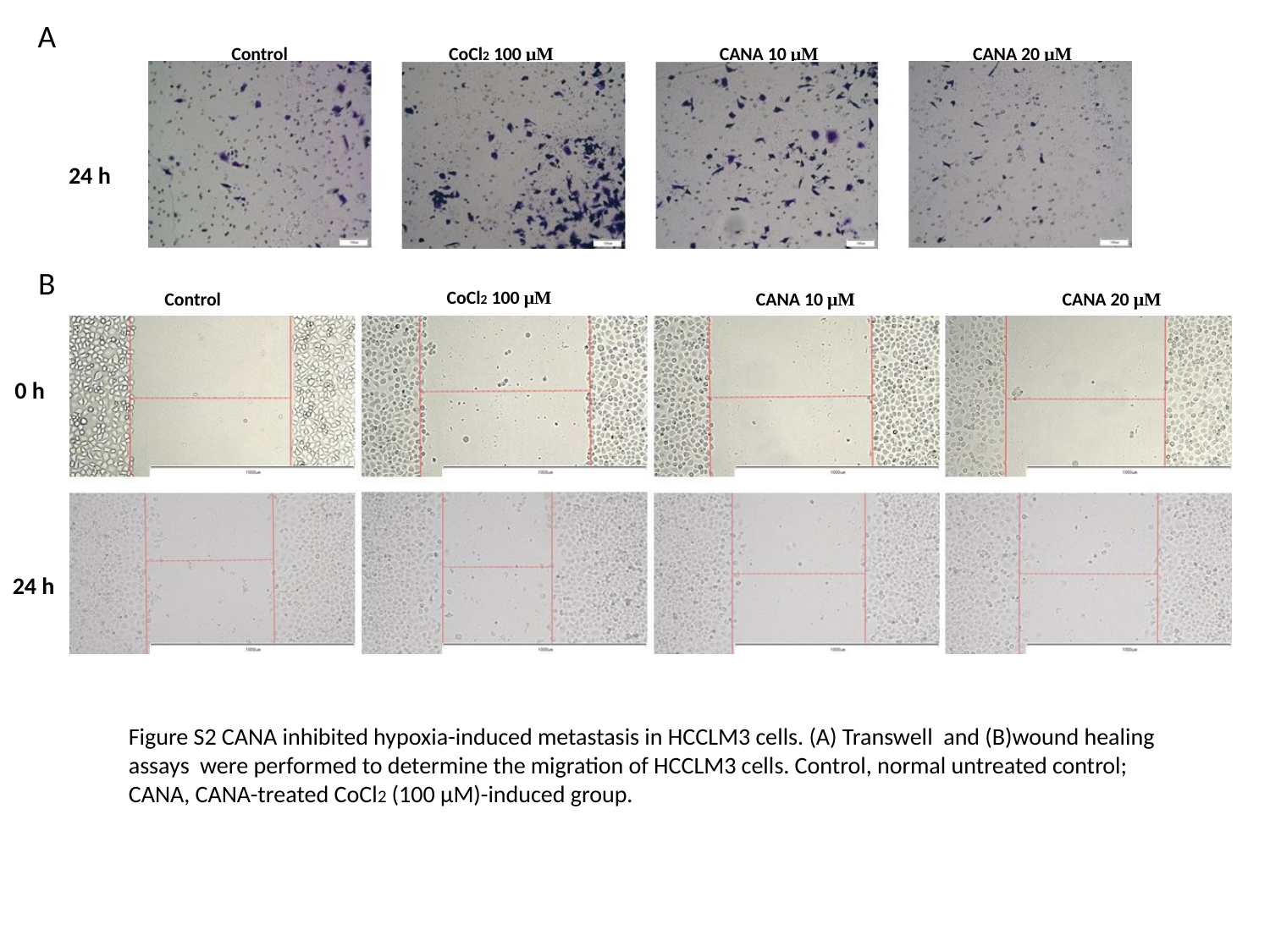

A
CANA 20 μM
CoCl2 100 μM
CANA 10 μM
Control
24 h
B
CoCl2 100 μM
Control
CANA 10 μM
CANA 20 μM
0 h
24 h
Figure S2 CANA inhibited hypoxia-induced metastasis in HCCLM3 cells. (A) Transwell and (B)wound healing assays were performed to determine the migration of HCCLM3 cells. Control, normal untreated control; CANA, CANA-treated CoCl2 (100 μM)-induced group.

## Slide 3
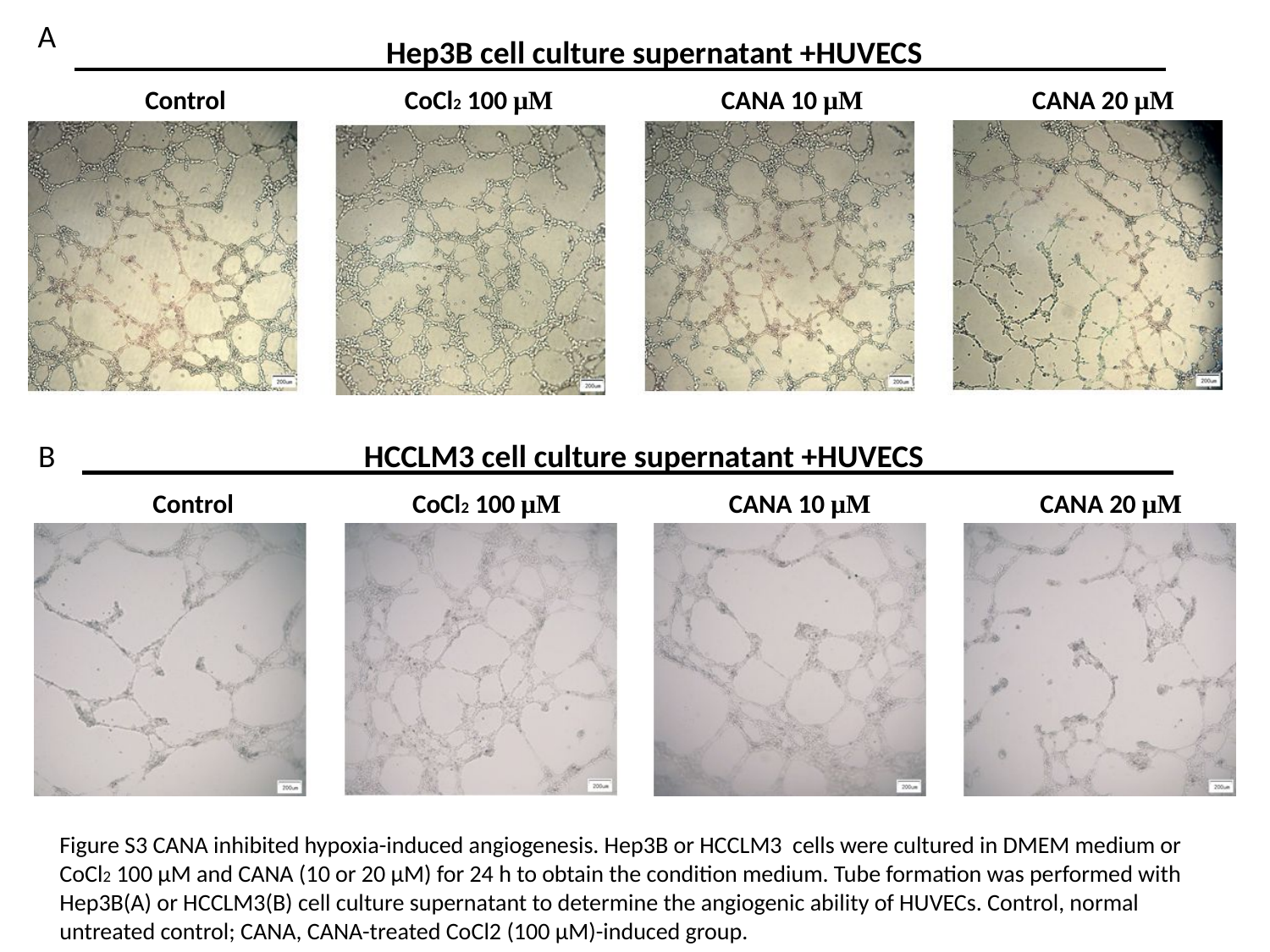

A
Hep3B cell culture supernatant +HUVECS
Control
CANA 10 μM
CANA 20 μM
CoCl2 100 μM
B
HCCLM3 cell culture supernatant +HUVECS
Control
CANA 10 μM
CANA 20 μM
CoCl2 100 μM
Figure S3 CANA inhibited hypoxia-induced angiogenesis. Hep3B or HCCLM3 cells were cultured in DMEM medium or CoCl2 100 μM and CANA (10 or 20 μM) for 24 h to obtain the condition medium. Tube formation was performed with Hep3B(A) or HCCLM3(B) cell culture supernatant to determine the angiogenic ability of HUVECs. Control, normal untreated control; CANA, CANA-treated CoCl2 (100 μM)-induced group.

## Slide 4
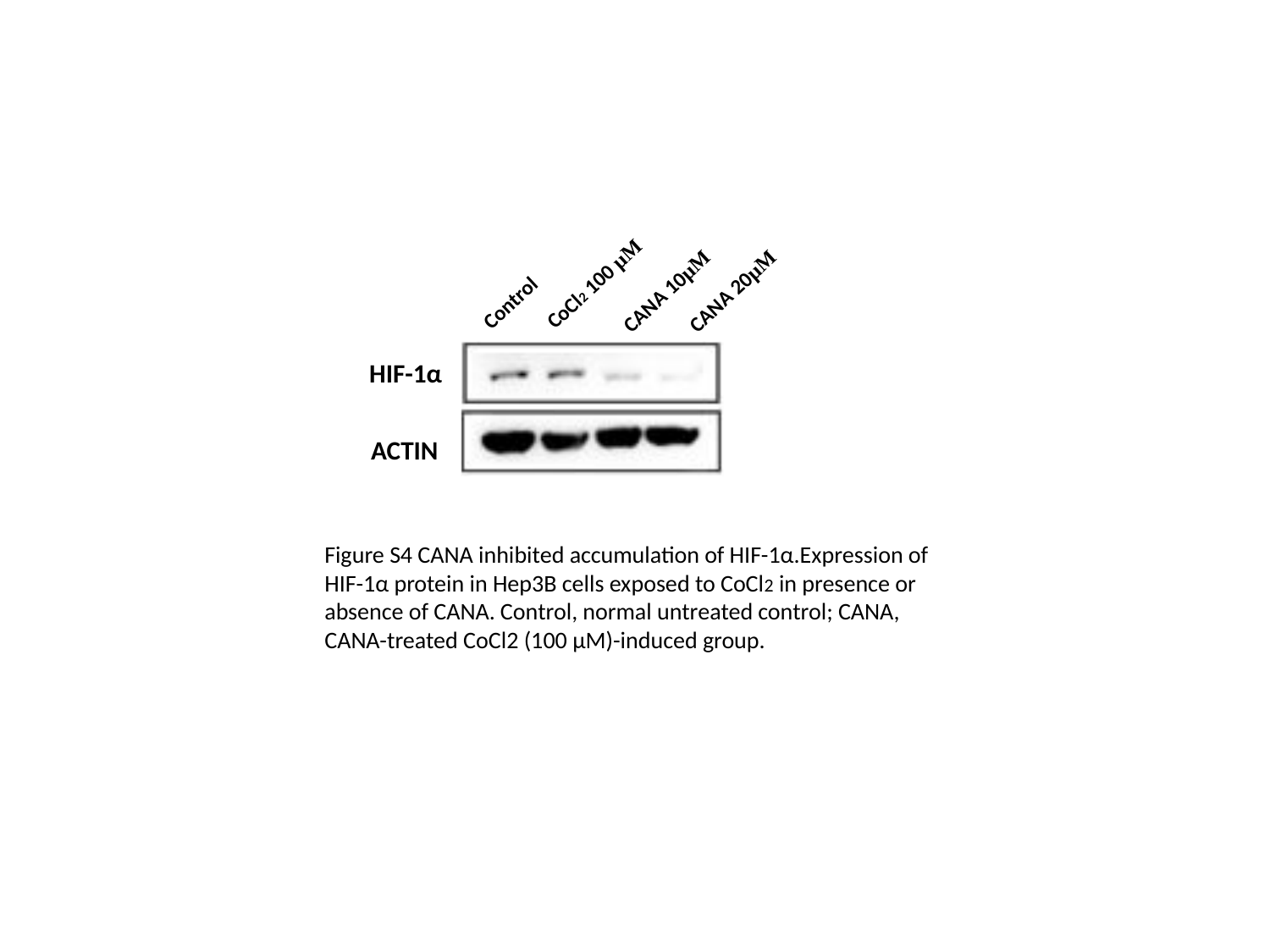

CoCl2 100 μM
CANA 10μM
CANA 20μM
Control
HIF-1α
ACTIN
Figure S4 CANA inhibited accumulation of HIF-1α.Expression of HIF-1α protein in Hep3B cells exposed to CoCl2 in presence or absence of CANA. Control, normal untreated control; CANA, CANA-treated CoCl2 (100 μM)-induced group.

## Slide 5
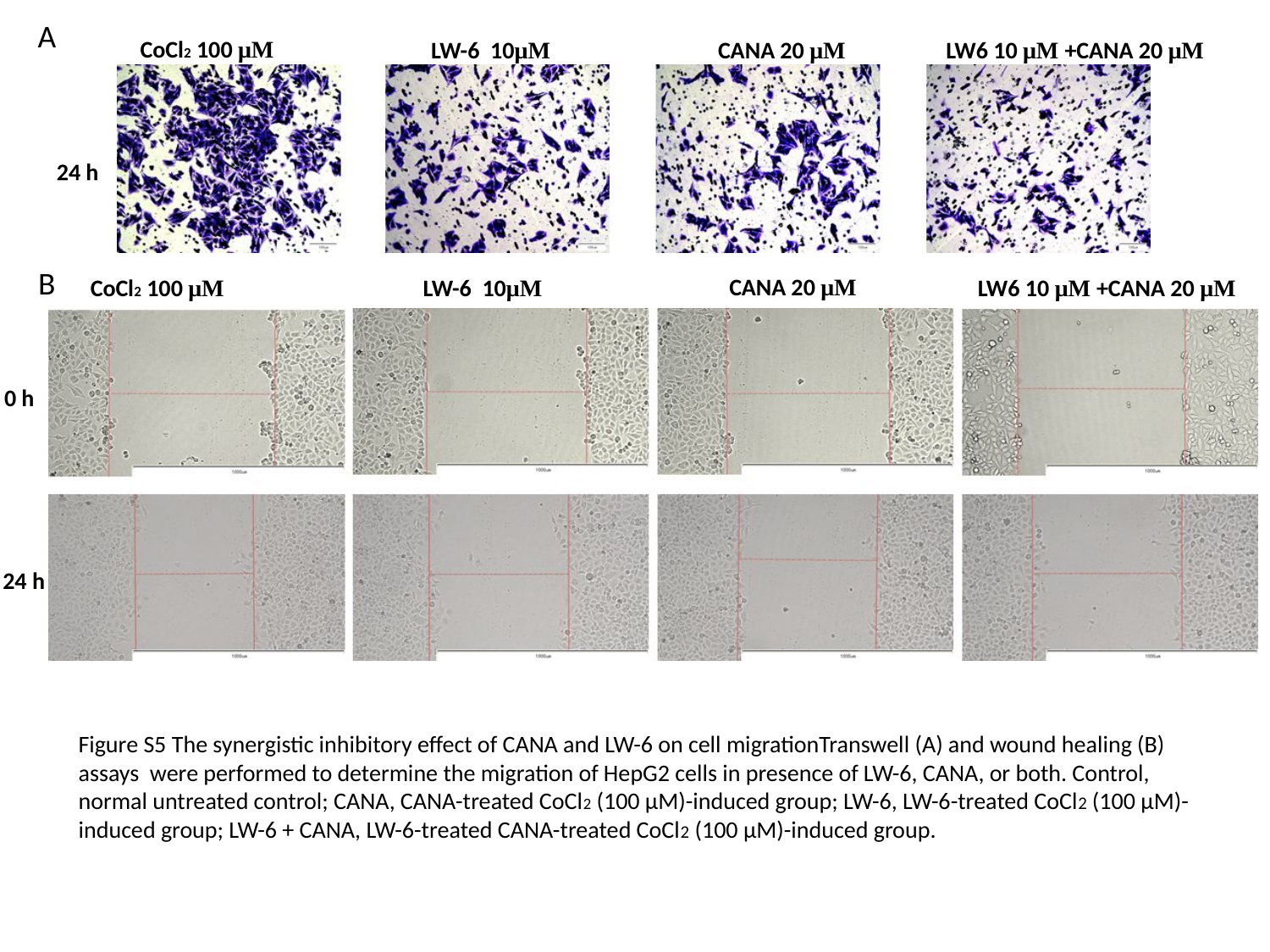

A
CoCl2 100 μM
LW-6 10μM
LW6 10 μM +CANA 20 μM
CANA 20 μM
24 h
B
CANA 20 μM
LW-6 10μM
CoCl2 100 μM
LW6 10 μM +CANA 20 μM
0 h
24 h
Figure S5 The synergistic inhibitory effect of CANA and LW-6 on cell migrationTranswell (A) and wound healing (B) assays were performed to determine the migration of HepG2 cells in presence of LW-6, CANA, or both. Control, normal untreated control; CANA, CANA-treated CoCl2 (100 μM)-induced group; LW-6, LW-6-treated CoCl2 (100 μM)-induced group; LW-6 + CANA, LW-6-treated CANA-treated CoCl2 (100 μM)-induced group.

## Slide 6
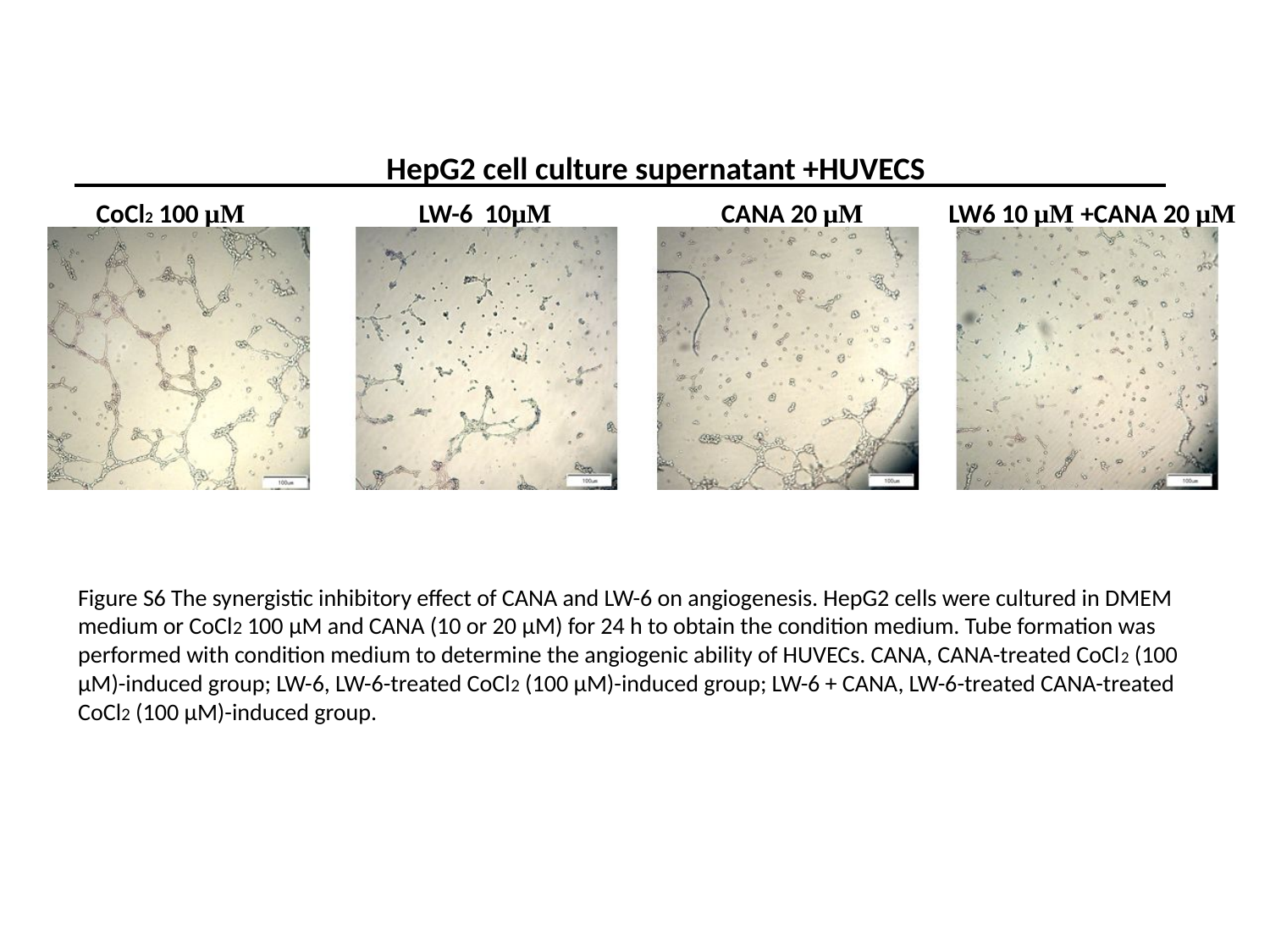

HepG2 cell culture supernatant +HUVECS
LW-6 10μM
CANA 20 μM
LW6 10 μM +CANA 20 μM
CoCl2 100 μM
Figure S6 The synergistic inhibitory effect of CANA and LW-6 on angiogenesis. HepG2 cells were cultured in DMEM medium or CoCl2 100 μM and CANA (10 or 20 μM) for 24 h to obtain the condition medium. Tube formation was performed with condition medium to determine the angiogenic ability of HUVECs. CANA, CANA-treated CoCl2 (100 μM)-induced group; LW-6, LW-6-treated CoCl2 (100 μM)-induced group; LW-6 + CANA, LW-6-treated CANA-treated CoCl2 (100 μM)-induced group.
